# Co-production of randomized clinical trials with patients: a case study in autologous hematopoietic stem cell transplant for patients with scleroderma

**DOI:** 10.1186/s13063-021-05575-0

**Published:** 2021-09-09

**Authors:** Magda Aguiar, Tracey-Lea Laba, Sarah Munro, Tiasha Burch, Jennifer Beckett, K. Julia Kaal, Nick Bansback, Marie Hudson, Mark Harrison

**Affiliations:** 1 Faculty of Pharmaceutical Sciences, University of British Columbia, 4625-2405 Wesbrook Mall, Vancouver, BC V6T 1Z3 Canada; 2 Centre for Health Economics Research and Evaluation, University Technology Sydney, Sydney, NSW Australia; 3 Centre for Healthcare Evaluation and Outcome Sciences, Vancouver, BC Canada; 4 Department of Family Practice, University of British Columbia, Vancouver, BC Canada; 5 Scleroderma Association of BC, Vancouver, BC Canada; 6 Patient Partner, Vancouver, Canada; 7 Patient Partner, Kamloops, Canada; 8 School of Population and Public Health, University of British Columbia, Vancouver, BC Canada; 9 Arthritis Research Canada, Richmond, BC Canada; 10 Division of Rheumatology, Jewish General Hospital and Lady Davis Institute, and Department of Medicine, McGill University, Montreal, QC Canada

## Abstract

**Background:**

Increasingly, it is argued that clinical trials struggle to recruit participants because they do not respond to key questions or study treatments that patients will be willing or able to use. This study explores how elicitation of patient-preferences can help designers of randomized controlled trials (RCTs) understand the impact of changing modifiable aspects of treatments or trial design on recruitment.

**Methods:**

Focus groups and a discrete choice experiment (DCE) survey were used to elicit preferences of people with scleroderma for autologous hematopoietic stem cell transplant (AHSCT) treatment interventions. Preferences for seven attributes of treatment (effectiveness, immediate and long-term risk, care team composition and experience, cost, travel distance) were estimated using a mixed-logit model and used to predict participation in RCTs.

**Results:**

Two hundred seventy-eight people with scleroderma answered the survey. All AHSCT treatment attributes significantly influenced preferences. Treatment effectiveness and risk of late complications contributed the most to participants’ choices, but modifiable factors of distance to treatment center and cost also affected preferences. Predicted recruitment rates calibrated with participation in a recent trial (33%) and suggest offering a treatment closer to home, at lower patient cost, and with holistic, multidisciplinary care could increase participation to 51%.

**Conclusions:**

Through a patient engaged approach to preference elicitation for different features of AHSCT treatment options, we were able to predict what drives the decisions of people with scleroderma to participate in RCTs. Knowledge regarding concerns and the trade-offs people are willing to make can inform clinical study design, improving recruitment rates and potential uptake of the treatment of interest.

**Supplementary Information:**

The online version contains supplementary material available at 10.1186/s13063-021-05575-0.

## Introduction

There is a long, established recognition that clinical research does not always translate to improvements in patient care and outcomes [1–3]. Randomized clinical trials are considered to be one of the most rigorous sources of evidence to evaluate treatments and services and should have the greatest potential to impact positively on the health care we provide to patients. However, it is argued that the majority of clinical trials are not useful since they do not respond to key questions [4], often fail to inform clinical decision-making [2], frequently fail to meet their recruitment targets [5], and contribute to an estimated 85% of wasted research spending [3, 4]. The proposed solutions to reducing waste and increasing value consistently include a greater role of patient preferences and priorities in the research process [1–5].

There has been a push to enhance involvement of patients in the research process, for example the National Institute for Health Research (NIHR) in the UK, Patient-Centered Outcomes Research Institute (PCORI) in the US, and Strategy for Patient Oriented Research (SPOR) in Canada [6–8]. Each requires active patient and public involvement (PPI) in the research they fund and promotes multiple methods to support inclusion of patients [9]. The role of and evaluation of impact of PPI in trials appears to focus on the process of enrolling and retaining potential participants; a recent review of the impact of PPI identified 26 clinical trials that used PPI to design strategies for recruitment and retention, patient information, and ways to identify and approach potential participants [10]. Organizations like the James Lind Alliance are improving understanding of patient research priorities in specific conditions or areas of health care [11], and pragmatic trials designs which reflect the real world are emerging [12, 13]. There has, however, been less attention on the role of PPI in co-production of research, which involves sharing power with patients from the point of generating the specific questions that the trial should answer or the design of the trial [14], and there is a lacuna of published methods to understand the extent to which trials of specific treatments and research questions are patient-centered and feasible in a target population. PPI in the design of trials has been consistently recommended, with specific reference to waste and inefficiency occurring due to choice of treatment and design of trials [15], and recognition that useful research should be patient-centered and “aligned with patient priorities, the utilities patients assign to different problems and outcomes, and how acceptable they find interventions” [2]. A recent paper proposed a role for using discrete choice experiments (DCE), a quantitative technique to elicit user preferences, in the design of complex interventions to promote higher uptake and adherence [16]. This type of approach could be used routinely at a formative stage of trial design to ensure that procedures, interventions, and outcomes are those that align with patient preferences and improves the likelihood of impact from research.

This paper describes a systematic approach to understand patients’ preferences to inform the design of a future clinical trial. It focuses on modifiable factors of the study or treatment (e.g., logistics, quality of care, and information provided), rather than non-modifiable factors (e.g., attitude to risk about an experimental treatment) [17]. Just as successful companies offer products and services that consumers want and need, a successful and useful trial should be investigating treatments and services that patients value and would be willing to use. By working with, listening to, and understanding patient preferences, we believe these methods could inform the design of treatments and services studied in RCTs, outcome measures, and the effect sizes needed and ultimately increase participation and retention rates of RCTs.

## Methods

### Case study

The “Scleroderma: Cyclophosphamide or Transplantation (SCOT)” trial, which tested autologous hematopoietic stem cell transplant (AHSCT) for people with scleroderma, is an example of a published trial which experienced difficulty in recruiting participants. The trial initially planned to recruit 226 participants to study event-free survival over 54 months [18]. However, slow recruitment led to a downward revision of the recruitment target to 114 participants, broadening of the entry criteria, and a change in the primary outcome measure to the global rank composite score, a hierarchy of 5 outcomes ranging from death to skin involvement [18]. Despite these changes, only 75 people were randomized (33%) due to “slow accrual” [18], which was later attributed to concerns about transportation and insurance coverage among potential participants, the latter being an important barrier to trial participation in the United States (US) [19].

### Approach

We conducted an online DCE survey to elicit the preferences of people with scleroderma for AHSCT treatment. DCEs were originally developed as a market research method to establish the value of goods and services, and in turn pricing, ahead of market launch, and have now been used in health economics for over 20 years. Preferences for new treatments and services are a natural application of this methodology because they are not yet available in routine patient care, and there is uncertainty about whether patients will be willing to use them. As such, quantitative methods to elicit patient preferences, such as DCEs, are recommended by the US Food and Drug Administration (FDA) as supplementary evidence to support decision makers achieving more patient-oriented decisions regarding trade-offs between benefits and risks [20].

### Patient and public involvement

A conversation between patients, clinicians, and researchers (TB, MHu, MH, NB) about the interpretation of the SCOT trial for patient-physician decision-making resulted in development of a patient-oriented project, to explore how patient perspectives could be formally elicited and used in design of RCTs. Two patient partners (TB, JB) were integrated into the research team and contributed to all stages of the project, including formulation of the research question and funding application (TB), survey design, recruitment, data analysis, interpretation, and dissemination (TB and JB); this process and the level of engagement aligns with the concept of co-production in PPI [14] which has been described elsewhere and our framework for patient involvement is summarized in the supplementary material (Table S1) [21, 22].

### DCE survey design

The survey consisted of four main sections: demographic information, information about AHSCT treatment (general information about eligibility and treatment including the potential process, risks and benefits), the DCE component, and questions about their health.

**Fig. 1:**
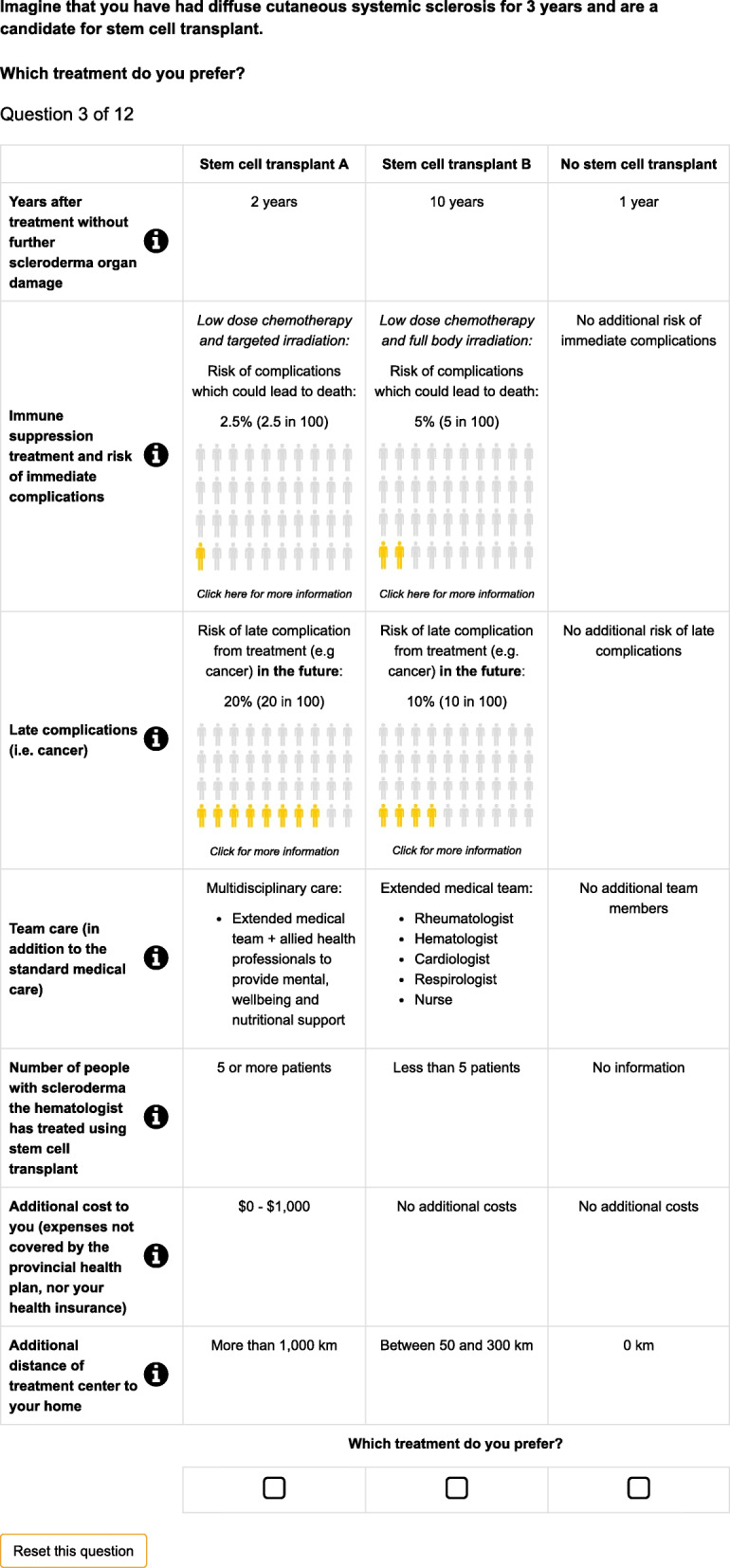
Example choice set presented to participants

The methods to develop the DCE followed published international guidelines for conducting DCEs in health [23–25] Participants were asked to choose between “AHSCT treatment A,” “AHSCT treatment B,” and a fixed “no AHSCT treatment” alternative. An example choice set is shown in Fig. 1. Treatment characteristics (attributes) were developed using qualitative methods [22], as recommended by best practice [24], and reflect the most important aspects of the decision about whether or not to undergo AHSCT treatment (Supplementary material (Table S1)). We used a nominal group technique (NGT) in a focus group with eight people with scleroderma in British Columbia, as part of a patient-oriented qualitative approach to design the DCE [22]. The NGT was designed to allow in-person and virtual participation to allow the perspectives of scleroderma patients in urban, rural and remote settings to participate. Participants were recruited via an email advert sent to all members of the Scleroderma Associations of British Columbia. The NGT approach had the advantage that it began with all potential attributes being generated by the patients participating in the group and finished with agreement on the most important features to be included as attributes. The NGT process was chaired by a qualitative researcher with experience in facilitating focus groups (SM) and a patient partner with lived experience of scleroderma on our research team (TB) and was completed within 2 h. The NGT process has been documented in detail elsewhere (Munro S, Aguiar M, Burch T, Kaal K, Trenaman L, Hudson M, et al. What are the factors that patients prioritise when considering novel treatments? A case study of systemic sclerosis using the nominal group technique. Submitted). Briefly, during the NGT process, participants considered what factors would matter to them if considering stem cell transplant for their scleroderma; each participant generated ideas independently, before all ideas were shared, recorded, and discussed. Once all ideas had been recorded and discussed, each participant independently voted on the five most important factors to them, and then shared these rankings to the rest of the group. Finally, the results were then discussed as a group.

The levels of each of the attributes were developed based on the literature, and the expert opinion of people with scleroderma (TB, JB) and clinicians (MHu) in the team, and qualitative data collected for this study. The final list of attributes and levels is presented in Table 1.

**Table 1:** Final list of attributes, levels, and data sources

Attribute	Levels	Sources/references
Years after treatment without further scleroderma organ damage	1. 1 year (opt-out level)	Expert opinion (clinician); Sullivan et al. [18]
	2. 2 years	
	3. 5 years	
	4. 10 years	
Immune suppression treatment and risk of immediate complications	1. No additional risk of immediate complications (opt-out level)	Carreras et al. [26]
	2. Chemotherapy; risk of complications which could lead to death: 10% (10 in 100)	
	3. Low dose chemotherapy and full body irradiation; risk of complications which could lead to death: 5% (5 in 100)	
	4. Low dose chemotherapy and targeted irradiation; risk of complications which could lead to death: 2.5% (2.5 in 100)	
Late complications (i.e., cancer)	1. No additional risk of late complications (opt-out level)	Refs [26–29]
	2. Risk of late complication from treatment (e.g., cancer) in the future: 5% (5 in 100)	
	3. Risk of late complication from treatment (e.g., cancer) in the future: 10% (10 in 100)	
	4. Risk of late complication from treatment (e.g., cancer) in the future: 20% (20 in 100)	
Team care (in addition to the standard medical care)	1. No additional team members (opt-out level)	Qualitative work
	2. Extended medical team: rheumatologist, hematologist, cardiologist, respirologist, nurse	
	3. Multidisciplinary care: extended medical team + allied health professionals to provide mental, wellbeing and nutritional support	
Number of people with scleroderma the hematologist has treated using AHSC treatment	1. No information (opt-out level)	Qualitative work/expert opinion (patient/clinician)
	2. Less than 5 patients	
	3. 5 or more patients	
Additional cost to you (expenses not covered by the provincial health plan, nor your health insurance)	1. No additional costs (opt-out level)	Qualitative work/expert opinion (patient)
	2. $0–$1,000	
	3. $1000–$5000	
	4. $5000–$10,000	
Additional distance of treatment center to your home	1. 0 km (opt-out level)	Qualitative work/expert opinion (patient)
	2. Between 50 and 300 km	
	3. Between 300 and 1000 km	
	4. More than 1000 km	

The combination of all levels in the DCE would result in a total of 9216 possible unique choice sets. We used Ngene software to create an experimental design optimized to combine the levels in as few choice sets as possible, selecting the choice sets that yield more information about participants’ choices [30]. This resulted in 24 different choice sets, which were divided into 4 blocks of 6. Each participant was randomized to one of 6 survey versions which contained two blocks of questions and therefore made 12 choices.

We pilot-tested the survey in think-aloud interviews with seven people with scleroderma to assess their interpretation of the questions and ability to complete the task. This piloting process led to minor changes to the survey, for example reducing the length and the complexity of instructions for clarity, providing an estimate of the time required to complete the survey (as well as emphasizing that respondents could leave and return to the survey), and adding a progress bar.

The survey was translated into French, using an approved translation service of the Scleroderma Patient-centered Intervention Network (SPIN) cohort, who routinely develop condition-specific surveys in French. English and French versions of the survey were distributed to the mailing lists of the SPIN patient cohort [31] and the Scleroderma Associations of British Columbia and Quebec in Canada in late 2019. Ethical approval was granted by the University of British Columbia behavioural ethics board (H18-02389).

### Statistical analysis

The DCE data was analyzed in STATA 15.6 software using a mixed multinomial logit (random parameters logit) model [32]. The MXL model accounts for preference heterogeneity in the population preferences by allowing selected parameter estimates (coefficients) to vary as random parameters rather than treating coefficients as fixed parameters. This infers that each participant in the sample has an individual-specific preference which leads to a specific parameter estimate on the distribution for each coefficient. Attribute levels were effects coded which enables the model to display coefficients for each single level per attribute. Effects coded coefficients are interpreted as relative preferences (with a central utility of 0) which are meaningless unless they are interpreted relative to the coefficients for the other attributes [33].

Predictions of recruitment to an RCT were calculated by first estimating the indirect utility of a specific AHSCT treatment option and no treatment, calculated as the sum of the coefficients for the levels of each attribute which best describes each scenario. The probability of participation in the trial was then calculated by dividing the exponential of the indirect utility of the AHSCT treatment by the sum of the exponential indirect utilities of AHSCT treatment and no treatment.

#### Understanding preferences

The model provides parameter estimates of the mean effect (coefficient) of each attribute level and the standard deviation of this parameter estimate for the population. The resulting coefficients indicate the relative importance of the levels of an attribute and the face validity of the results. The bigger the coefficient, the greater the importance of the attribute level for the decision. Positive coefficient indicates that respondents attach a positive value to that particular level, while a negative coefficient indicates indicate a negative value to that level. Higher risks of adverse events, for example, are expected to have negative coefficients.

#### Using preferences to predict participation in a trial

The inclusion of the fixed “no AHSCT treatment” opt-out alternative allows uptake to be predicted [34]. The coefficients for each attribute level can be used to estimate the utility (or value) of a treatment, based on the levels of each attribute which best describe that treatment. As a test of the external validity of our results, we predicted the uptake for the SCOT trial, based on estimates of preferences for treatment from the DCE results (stated preferences), and compared this with the observed 33% (75/226) participation rate of the SCOT trial (revealed preference). Levels for the SCOT trial were chosen in consultation with people with scleroderma and clinicians in our team and the trial publication (Table 2) [18].

**Table 2:** Description of the AHSCT treatment interventions

	Original trial design	Patient-oriented trial design
Years after treatment without further scleroderma organ damage	1–10 years	1–10 years
Members of the care team	Extended medical team: rheumatologist, hematologist, cardiologist, respirologist, nurse	Multidisciplinary care: extended medical team + allied health professionals to provide mental, wellbeing and nutritional support
Immediate complication	Low dose chemotherapy and full body irradiation; risk of complications which could lead to death: 5% (5 in 100)	Low dose chemotherapy and full body irradiation; risk of complications which could lead to death: 5% (5 in 100)
Late complications	Risk of late complication from treatment (e.g., cancer) in the future: 20% (20 in 100)	Risk of late complication from treatment (e.g., cancer) in the future: 20% (20 in 100)
Hematologist’s experience	5 or more patients	5 or more patients
Additional cost to you	$1000–$5000	$0 to $1000
Distance of the treatment center from your home	More than 1000 km	Between 50 and 300 km

We then predicted the potential impact on participation in a trial which offered a treatment whose modifiable attributes (cost, distance, team care) were adapted to be more aligned with preferences of people with scleroderma (made more favorable by one level).

## Results

### Sample

Two hundred seventy-eight people with scleroderma (71%) completed the survey (out of 389 who started the survey) (Table 3). The majority of the sample identified as women (88%), were aged 40 years or older (90%), Caucasian (74%), and lived in Canada (45%), the USA (28%), or France (17%). Over half of the sample (54%) had diffuse scleroderma, the main diagnosis for which AHSCT treatment is currently indicated, 44% had limited scleroderma, and the remaining 3% reported other types of scleroderma. Disease duration ranged from 0 to 54 years (mean 13.9 year, SD 9.9 years) and 51% of the respondents reported being 40–59 years old at diagnosis. Disease duration was longer for those with limited scleroderma (16.0 years, standard deviation 11.4 years) than those with diffuse scleroderma (12.3 years, standard deviation 8.3 years) (*p* = 0.002). Of those with diffuse scleroderma, 19 (13%) people had disease duration of less than 5 years, which is broadly representative of the potential candidates for AHSCT treatment.

**Table 3:** Participant characteristics

		*n* = 278	Percent
Age	18–39 years old	27	10
	40–59 years old	120	43
	60+	131	47
Gender	Woman	244	88
	Man	32	12
	Gender fluid, non-binary, and/or Two-Spirit	1	< 1
	Prefer not to say	1	< 1
Scleroderma type	Limited	121	44
	Diffuse	150	54
	Other	7	3
Age at diagnosis	0–18 years old	8	3
	18–39 years old	94	34
	40–59 years old	141	51
	60+	35	13
Disease duration	0–4 years	37	13
	5–9 years	77	28
	10–19 years	99	36
	20–29 years	38	14
	30+ years	27	10
Country	Canada	123	44
	USA	77	28
	France	49	18
	UK	21	8
	Côte d’Ivoire	1	< 1
	Zambia	1	< 1
Identification*	Aboriginal or indigenous	22	8
	African American or Black	8	3
	Asian	7	3
	Caucasian	206	74
	Hispanic or Latino(a)	10	4
	South Asian	3	1
	How I identify is not listed here	23	8
	Prefer not to say	4	1

*Respondents could choose multiple categories

### Preferences of people with scleroderma

#### Face validity

**Fig. 2:**
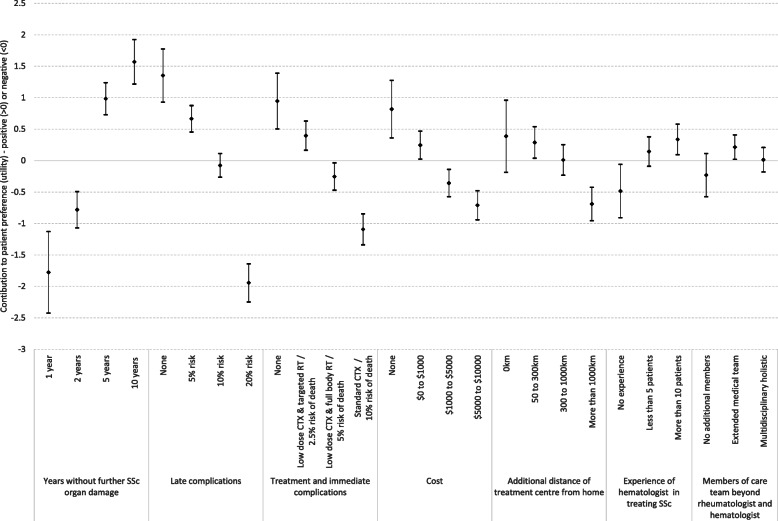
Preferences of people with scleroderma for aspects of stem-cell transplant treatment

Figure 2 shows that estimated preferences for attribute levels were ordered as expected, supporting the face validity of the survey. For example, larger benefits (e.g., 5 or 10 years without further organ damage) from treatment contributed positively to preferences, while small benefits (e.g., 1 or 2 years without further organ damage) contributed negatively. Similarly, lower levels of risks, costs, and travel distance contributed positively to preferences, while higher levels of these characteristics contributed negatively

#### Preferences for aspects of AHSCT

The most important characteristics of a decision to undergo AHSCT, or not, were the potential risks and benefits; the highest levels of the risk of either the immediate or late complications would either completely (late complications) or partially (immediate complications) offset preferences for the highest level of benefit (10 years without further organ damage). The cost of treatment to an individual was a priority for people with scleroderma; the magnitude of importance of cost to individuals was only slightly smaller than the risk of immediate complications from treatment. Distance was not statistically significant until it exceeded 1000 km. The experience of the transplant hematologist in treating people with scleroderma was a priority only at very low (negative preference) or high levels (positive preference). There was a preference for additional members of the care team, but this was only small and statistically significant for an extended medical team.

### Predicting trial participation based on preferences for aspects of AHSCT

#### External validity

**Fig. 3:**
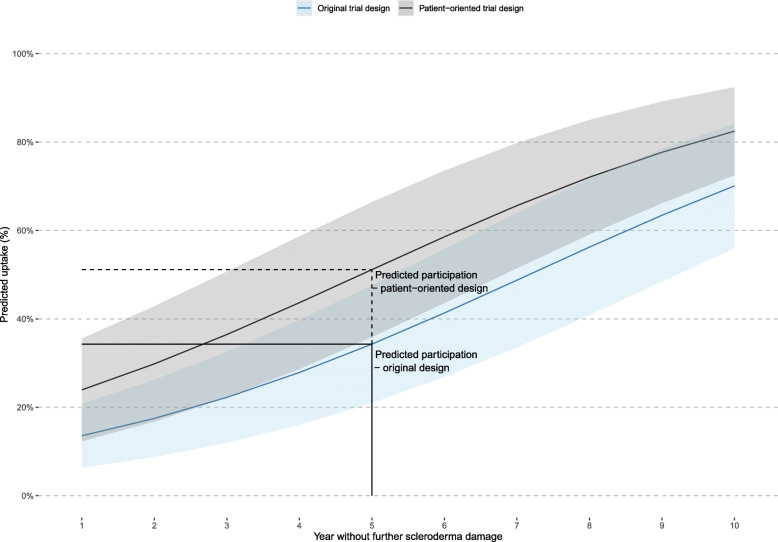
Predicted uptake of treatment based on preferences for autologous hematopoietic stem cell transplant for scleroderma processes and outcomes

Using preferences for attribute levels from our model, at an expected benefit of 5 years without further organ damage, which most closely matches the intended SCOT trial primary outcome, we predict that around 1 in 3 people (34%) with scleroderma would be willing to participate in a trial of this treatment (shown in blue, Fig. 3). This corresponds very closely with the reported participation in the SCOT trial, which recruited 33% of the target sample size; this offers evidence of the external validity of our predictions.

**Fig. 4:**
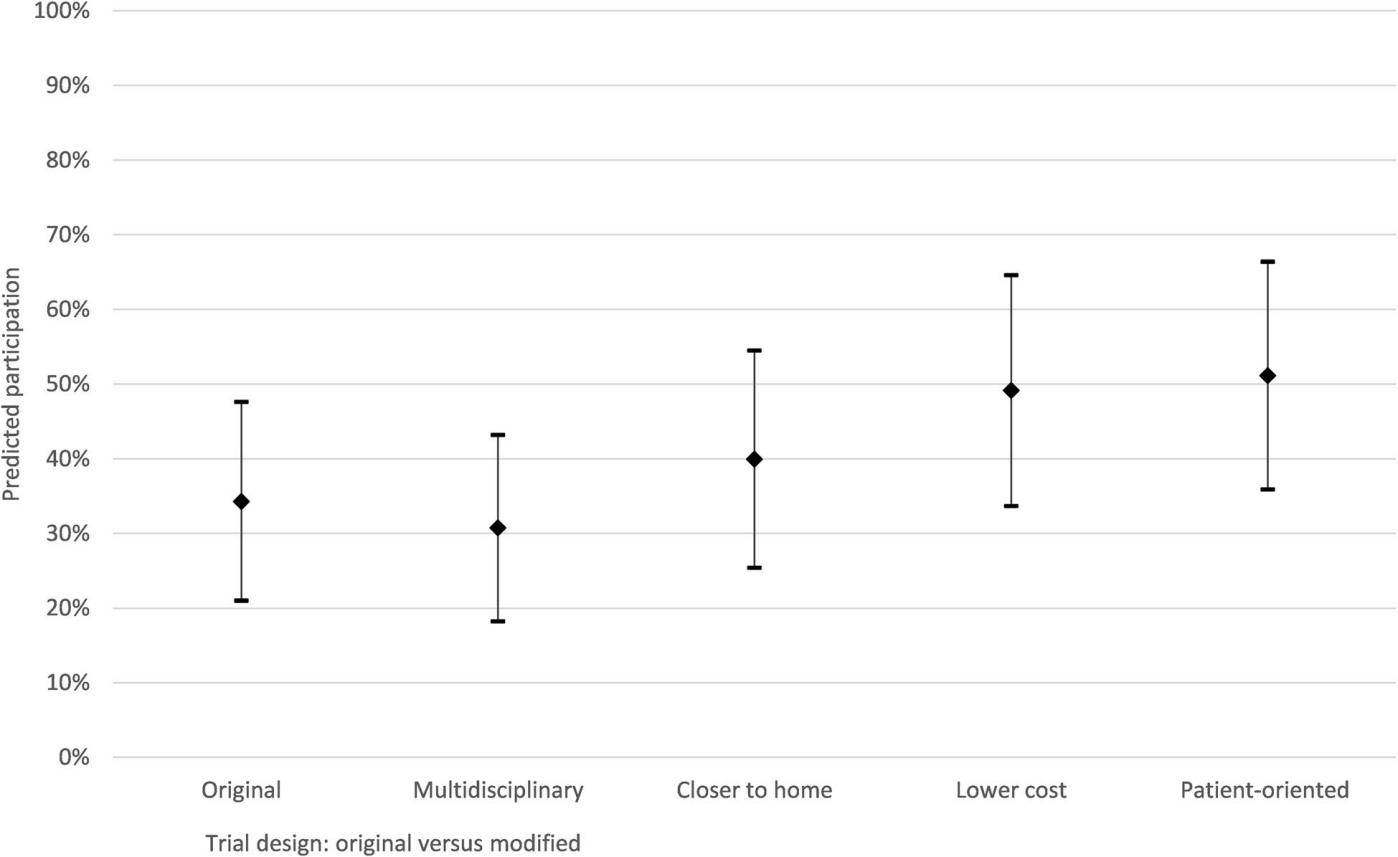
Predicted uptake of the treatment before and after modifications based on the preferences of people with scleroderma

Figure 3 shows the potential impact of preferences for a treatment on participation in an RCT of changing modifiable factors in the delivery of AHSCT to make the design more “patient-oriented,” broadening the team involved in care (by adding multidisciplinary care), offering treatment closer to home (< 300 km), and reducing costs to individuals ($0–$1000). Predicted participation, for a treatment with the same risks and benefits, could be increased to 51% with all modifications. The potential contribution of each modifiable factor is shown in in Fig. 4; offering a treatment closer to home and at a lower cost appears to most influence the likelihood of participation.

## Discussion

This study revealed the preferences people with scleroderma have for potentially modifiable aspects of AHSCT, how changes to these aspects could make the treatment more acceptable, and in turn a trial of the treatment more appealing to participate in. Understanding whether a treatment is acceptable to the people it is intended for has important implications in deciding whether or not a treatment should be studied in a RCT and whether patients will participate in a trial. Our results revealed that people might be willing to trade key RCT outcomes (efficacy and risks) for improvements in some modifiable procedural factors (costs, distance of the treatment center to their home, care team characteristics), highlighting that designing treatments that align with patient preferences could improve participation in RCTs. There are other factors involved in choosing to participate in a trial, including the chance of being randomized to a control arm, the uncertain effects of the treatment [35, 36], but understanding whether the proposed treatment is valuable for a patient is a natural first step.

Our findings align with the literature that suggests that other factors beyond clinical outcomes influence people’s willingness to participate in RCTs [37]. Our study expands on this knowledge by demonstrating how DCEs, a quantitative preference-based method, can be used to formally elicit and incorporate the preferences of patients into the design of RCTs. Such an approach has the potential to maximize the value of using PPI in research by involving patients from the early stages of the study and designing surveys which can gather the perspectives of larger, representative groups of patients in a relatively quick manner. If this type of pre-trial investigation is implemented, sub-group analysis could be done to understand preferences of particularly hard-to-recruit groups, such as minorities, and enhance success of achieving more diverse participation in RCTs.

Moreover, incorporating patient preferences in early stages of the RCT’s design might contribute to increase the sustainability of clinical research by reducing avoidable waste. There is concern about the extent of wasted investment in biomedical research and that promising research findings too frequently do not enter routine clinical practice or translate to improvements in health care [4]. Among the key stages that lead to wasted research investment is a failure to address questions and interventions that are most relevant to clinicians and patients and evaluating these questions using outcomes that are not the most important or relevant to patients [1]. Research waste may thus be explained by patients not wanting the treatment and services being researched.

DCEs are a well-established methodology in marketing and health economics research. This study further shows the potential value of the DCE methodology in the context of RCT design. First, it indicates the potential to improve recruitment rates by aligning modifiable process characteristics of the treatment or intervention to be studied in a trial with patient preferences. This potential is supported by the close alignment of our predicted uptake based on the preferences of people with scleroderma (34%), with the actual recruitment rate reported in the SCOT trial, which was able to recruit only 33% of their target sample size [18]. This is consistent with other studies which support the external validity of DCEs [38]. Furthermore, the investigators of this trial listed potential modifiable factors (transportation and insurance coverage [18]) which align with key modifiable factors that affect patient preferences in our study (cost and distance). Secondly, by replicating decision-making, which requires trade-offs to be made between features of treatments, these methods can reveal whether potential modifications to treatments will influence participation in a RCT. For example, we found that a holistic, multidisciplinary, team, which was strongly endorsed as an important theme by people with scleroderma in our qualitative work [22], did not positively impact predicted participation in a trial. We believe our results will be informative for the design of any future trials of AHSCT for people with scleroderma, as we have described the preferences and trade-offs for the delivery of this treatment using a large, diverse sample of people with scleroderma. However, our goal was also to use this case study as an example of how choice-based methods could be used to inform the design of future RCTs. The power of choice-based methods are that they help to isolate which factors are most important in influencing decision-making (or most valued by individuals) by presenting situations where multiple desirable aspects of a treatment must be traded off against each other [39]. We believe that this approach has applicability to both chronic and acute conditions. While the options for varying preferences in clinical practice may be more limited for acute or time-limited conditions as compared to chronic conditions, acute conditions are still preference-sensitive, i.e., requiring some form of trading between risks and benefits as well as other aspects of care that contribute to overall quality of care [40–42]. Because the opportunity to incorporate patient preferences for those other aspects of quality care for acute conditions is limited at the point of care, then one may argue that using preference methods to inform the design of treatments for acute conditions may be more pertinent than chronic conditions. Notwithstanding this, we acknowledge that those modifiable aspects of treatment which extend beyond risks and benefits but comprise quality care are likely different for patients with acute and chronic diseases.

The interpretation of the results must consider that the DCE developed in this study looked at preferences for alternative hypothetical scenarios (stated preferences), and it is not known how these will match the actual choices (revealed preferences) if the alternatives were to be available to patients. Nonetheless, our model and predictions have shown favorable internal and external validity. Furthermore, this study was both patient-oriented, including people with scleroderma as patient partners on the research team from the conception of the study idea, to dissemination of results, and followed best practice recommendations to identify all relevant treatment attributes through qualitative research methods. Finally, we acknowledge that while the SCOT trial, which we use as the case study for this paper, might not provide definitive information to inform patient-physician decision-making, it does represent an important step forward in treatment options for people with severe scleroderma.

The feasibility of using methods like DCEs to understand patient preferences in the design phase do warrant consideration—there are concerns about the time and funds required to conduct DCEs and that the results may have limited predictive ability. Additionally, our DCE predicts participation in a RCT based on preferences for a treatment, but not preferences to participate in a trial which offers a chance of being randomized to a treatment. Considering these in order, using DCEs in the design phase could add considerable time to the process; however, it is worth noting that it is the time required to design a DCE (identifying key attributes and their levels) and collect responses that is the time-consuming component; the analysis and interpretation of data is relatively quick. In case of proposed RCTs, however, the non-modifiable attributes are likely to be known and the potentially modifiable could be identified as part of the PPI that is recommended as part of good trial design [6–9]. Approaches like the nominal group technique we used in this study could be formally conducted as part the process of PPI and could quickly identify the priorities of patients for aspects of treatment. Our study demonstrates that this NGT in itself can be valuable as part of a patient-oriented approach, as well as directly identifying key attributes for a DCE. The requirement for funding to conduct a DCE as part of the design phase of RCTs is also not a unique barrier; RCTs often require funded feasibility and pilot studies to test trial design and aspects of recruitment, and a DCE could be rolled into data collection questionnaires and presented as part of preliminary work required as components of an application for funding the full RCT, just as sample size calculations are. Finally, the validity of predictions is a legitimate concern—the disconnect between stated (hypothetical) and revealed (actual) preferences (hypothetical bias) is well documented [43], but a recent review and meta-analysis found that stated preferences from DCEs can offer reasonable predictions of subsequent health-related behaviors, while cautioning a risk of over-predicting demand [44]. However, as long as any overprediction is consistent across all estimates of participation in a trial, the results should RCTs closer to the designs that reflect treatments that patients want and would use. Furthermore, it is unlikely that the incorporation of the preferences of larger, more representative samples of potential trial participants is likely to be detrimental in the design of RCTs. Finally, we recognize that our DCE sought preferences which allowed us to predict preferences for a treatment that could be offered in an RCT and not preferences for a treatment offered in the context of an RCT, i.e., subject to randomization. The additional layer of uncertainty about the treatment they would receive through randomization is therefore not considered in our predictions. Other studies seeking to explore how preferences for treatment alongside aspects of RCT design could inform people’s decision about whether or not to participate, also using DCEs [45]. However, while these studies complement our approach, we believe that before deciding to design an RCT, the first step is to determine whether the treatment you would study is desirable and acceptable to people you would recruit.

We propose that early studies, designed in collaboration with patients and using methods such as DCEs, should be a key part of designing RCTs to ensure that scarce research resources are spent only on interventions which address genuine patient needs, offer acceptable and usable solutions, and deliver outcomes that are valued by the people who matter—patients.

## Supplementary Information

**Additional file 1: Table S1.** The level of involvement of patient-partners throughout this project.

## Data Availability

The datasets used and/or analyzed during the current study are available from the corresponding author on reasonable request.
