# Supplementary material for: Co-production of randomized clinical trials with patients: a case study in autologous hematopoietic stem cell transplant for patients with scleroderma
Source: Trials. 2021 Sep 9;22:611. doi: 10.1186/s13063-021-05575-0 (PMC8428135; doi:10.1186/s13063-021-05575-0)
Supplement: Supplementary file 1 — Additional file 1: Table S1. The level of involvement of patient-partners throughout this project. [file 13063_2021_5575_MOESM1_ESM.docx]

**Supplementary material**

**Patient-involvement approach**

Table S1. The level of involvement of patient-partners throughout this project

|  | Patient-partner involvement: | | Level of involvement(43) | Supporting references |
| --- | --- | --- | --- | --- |
|  | TB | JB |  |  |
| Identifying research topic | Y |  | Co-creation |  |
| Application for funding | Y |  | Co-creation |  |
| Ethics application | Y |  | Co-creation |  |
| **Phase 1: DCE development study (qualitative)** |  |  |  |  |
| Design of qualitative study | Y | Y | Collaboration |  |
| Development of materials and participation information | Y | Y | Collaboration |  |
| Recruitment | Y |  | Involve |  |
| Facilitation of nominal group techniques | Y | Y | Involve |  |
| Analysis | N |  |  |  |
| Interpretation of results | Y | Y | Collaboration |  |
| **Phase 2: DCE study** |  |  |  |  |
| Design of DCE attributes, levels, wording, additional questions | Y | Y | Empower/Collaboration/Consulting |  |
| Recrutiment strategy | Y |  | Collaboration |  |
| Design of analysis | Y |  | Collaboration |  |
| Analysis of results |  |  |  |  |
| Interpretation of results | Y | Y | Collaboration |  |
| Drafting manuscripts | Y | Y | Collaboration |  |
| **Dissemination:** |  |  |  |  |
| Presentations at scientific meetings | Y |  | Empower | (44,45) |
| Presentations to patient organizations | Y |  | Empower | (46) |
| Patient perspective publication | Y |  | Empower | (20) |
| Patient involvement publication | Y |  | Empower | (20,21) |
| Main results paper | Y | Y | Empower |  |
| Financial compensation: | Y | Y |  |  |

**Additional references**

44. Burch T, Laba T-L, Beckett J, Aguiar M, Munro S, Kaal K, et al. Perspectives of shared decision-making with patient partners in the design and development of a scleroderma patient-centered research project in British Columbia [Internet]. Oral presentation presented at: 10th International Shared Decision Making Conference; 2019 Jul 7; Quebec City, Canada. Available from: https://fourwaves-sots.s3.amazonaws.com/static/media/uploads/2019/06/28/isdm2019-oralsessionsbooklet-2019-06-28.pdf

45. Aguiar M, Munro S, Burch T, Beckett J, Kaal K, Laba T-L, et al. Considering shared decision-making in the design of clinical studies: methodological insights on approaching people with scleroderma in British Columbia [Internet]. Oral presentation presented at: 10th International Shared Decision Making Conference; 2019 Jul 9; Quebec City, Canada. Available from: https://fourwaves-sots.s3.amazonaws.com/static/media/uploads/2019/06/28/isdm2019-oralsessionsbooklet-2019-06-28.pdf

46. Burch T, Harrison M. Identifying patient’s preferences when considering stem cell transplant as a treatment option for Scleroderma. Scleroderma Association of British Columbia Annual General Meeting and Conference; 2019 Oct 5; Coquitlam, BC, Canada.
